# Diagnosis of chronic conditions with modifiable lifestyle risk factors in selected urban and rural areas of Bangladesh and sociodemographic variability therein

**DOI:** 10.1186/1472-6963-11-309

**Published:** 2011-11-11

**Authors:** John D Parr, Wietze Lindeboom, Masuma A Khanam, Tracey L Pérez Koehlmoos

**Affiliations:** 1 International Center for Diarrheal Disease Research, Bangladesh, Health Systems and Infectious Disease Division. Mohakali, Dhaka Bangladesh

## Abstract

**Background:**

Bangladesh suffers from a lack of healthcare providers. The growing chronic disease epidemic's demand for healthcare resources will further strain Bangladesh's limited healthcare workforce. Little is known about how Bangladeshis with chronic disease seek care. This study describes chronic disease patients' care seeking behavior by analyzing which providers diagnose these diseases.

**Methods:**

During 2 month periods in 2009, a cross-sectional survey collected descriptive data on chronic disease diagnoses among 3 surveillance populations within the International Center for Diarrheal Disease Research, Bangladesh (ICDDR, B) network. The maximum number of respondents (over age 25) who reported having ever been diagnosed with a chronic disease determined the sample size. Using SAS software (version 8.0) multivariate regression analyses were preformed on related sociodemographic factors.

**Results:**

Of the 32,665 survey respondents, 8,591 self reported having a chronic disease. Chronically ill respondents were 63.4% rural residents. Hypertension was the most prevalent disease in rural (12.4%) and urban (16.1%) areas. In rural areas chronic disease diagnoses were made by MBBS doctors (59.7%) and Informal Allopathic Providers (IAPs) (34.9%). In urban areas chronic disease diagnoses were made by MBBS doctors (88.0%) and IAP (7.9%). Our analysis identified several groups that depended heavily on IAP for coverage, particularly rural, poor and women.

**Conclusion:**

IAPs play important roles in chronic disease care, particularly in rural areas. Input and cooperation from IAPs are needed to minimize rural health disparities. More research on IAP knowledge and practices regarding chronic disease is needed to properly utilize this potential healthcare resource.

## Background

Early identification is widely recognized as a crucial first step in proper chronic disease management. Many of the diseases are clinically silent for years and when symptomatic, can be difficult to manage [1]. Efforts to control chronic disease epidemics in developed countries focus on early diagnosis and disease screening [2,3] in order to prevent sequela of unmanaged disease (ie stroke, kidney failure, myocardial infarction).

Unmanaged chronic disease contributes to disability adjusted life years, straining the workforce and economy [4-6]. Unfortunately, chronic disease prevalence is steadily increasing worldwide, even among working age populations [4-7].

In 2002, chronic disease sequela comprised 44% of Bangladeshi mortalities [7]. In Matlab Bangladesh, noncommunicable disease mortality (excluding injury and accident) increased from 8% (1986) to 68% (2006) [8]. Such a shift, partially attributable to infectious disease oriented public health campaigns, places strain on healthcare systems designed to manage infectious disease. In Bangladesh, hypertension prevalence estimates are as high as 18% [9], although recent unpublished data suggest even higher rates. By 2025 an estimated 7.4 million Bangladeshis will have diabetes; currently at least 63% of Bangladeshi diabetics are estimated to be younger than 55 years old [10]. This shift in disease burden has serious implications for Bangladesh's economy, healthcare system and society; mitigating its impact will require a large health work force.

Shortages of MBBS doctors, coupled with limited training capacity, leave Bangladesh without an adequate healthcare workforce in the public sector [11,12]. Most Bangladeshis rely on the informal sector, particularly informal allopathic practitioners (IAP) (medicine shopkeepers and village doctors) for first line health care [11-15]. Many developing countries, including Bangladesh, recognize MBBS doctors as the highest level of care, but consider them inaccessible [16-18]. Accessibility and care seeking are influenced by cultural biases, education, economics, gender, and transportation constraints [19-22].

While care seeking data is crucial to shaping effective health policies, studies and initiatives aimed at addressing chronic diseases are still relatively new. There is a paucity of data on chronic disease care seeking behavior in Bangladesh [8,23-25]. This study seeks to compare urban and rural Bangladesh by examining prevalence of self reported chronic disease diagnoses, sources of chronic disease diagnoses and sociodemographic variations among care seeking groups.

## Methods

The study population was obtained from Health Demographic Surveillance System (HDSS) study populations at 3 sites; two rural, Abhoynagar (southwestern Bangladesh) and Mirsarai (southeastern Bangladesh) and one urban, Kamalapur (southeastern Dhaka). The total surveillance population in Abhoynagar in 2009 was 34,717. Abhoynagar's average household income was 5,609 Bangladesh Taka (BDT) per month (in 2008), median 4,000 BDT. Abhoynagar's disease profile is predominantly fever, digestive disturbance, and respiratory disease. The total surveillance population in Mirsarai in 2009 was 39,025. Mirsarai's average household income was 8,040 BDT per month (in 2008) median 6,000 BDT. Mirsarai's disease profile is predominantly fever, digestive disturbance, and respiratory disease [26]. Total surveillance population in Kamalapur was 32,441 (Kamalapur). On average, one quarter of Kamalapur residents live below the poverty line, with a monthly income (in 2009) less than 13,902 taka per month. HDSS Kamalapur disease profile data is currently unavailable [27]. Ethical clearance for surveillance site activities was obtained from the International Center for Diarrheal Disease Research, Bangladesh (ICDDR, B) review board.

Using a cross sectional survey, respondent data was collected for a 2 month period at each site, beginning in January 2009 and finishing in December 2009 (Additional File 1). The study population was limited to men and women over 25 years of age residing in a HDSS surveillance area. Information was collected during daytime household visits; only those present for the interview were included and a single interview attempt was made per household. Rural site data was collected during regular surveillance rounds. Urban site data was collected after the initial HDSS census. To detect the most prevalent chronic disease diagnosis, hypertension, at a maximum prevalence rate of 22% +/- 2.5% in each of the 4 age groups, 10,740 respondents (3,580 per site) were needed. The survey was part of routine surveillance making the actual sample size (32,665) equal to the maximum number of surveillance households that the HDSS surveillance teams could survey in that two month period. As a sample was not drawn from our defined HDSS surveillance population, non-response rate was not tracked. However, typical estimates of HDSS surveillance sites place absenteeism of a given household at less than 5% at the time of survey.

Individual socio-demographic factors were derived from preexisting surveillance data. Household based poverty quintiles approximated respondents' socio-economic status. Poverty quintiles were based on household assets (mobile phone, furniture, vehicle, appliances etc...) and housing characteristics (construction materials, energy used for cooking, ownership of household assets, income ect...). Further details have been published elsewhere [26,27]. Variable reduction techniques combined assets and household characteristics into a single asset variable [28]. After ranking this variable from low to high, households were divided into 5 equal sized poverty quintiles. This procedure was repeated for each site; household stratification did not account for possible poverty/wealth differences between sites.

Trained research assistants conducted the interviews in Bangla using a two part questionnaire on chronic disease lifestyle risk factors and management. Respondents were asked "Have you ever been told by any of the following personnel: MBBS doctor, specialized doctor, nurse, health worker, paramedic (Medical Assistant/Sub-Assistant Community Medical Officer), village doctor/quack, homeopath, kabiraj, or pharmacy man that you have any of the following medical conditions: hypertension, diabetes, abnormal blood lipids, overweight, chronic bronchitis, heart attack, angina/coronary heart disease, stroke, asthma, oral cancer, lung cancer, other." Respondents were then asked to identify the type of provider that diagnosed their condition most recently. The disease categories were selected by a panel of experts for their high prevalence, modifiable lifestyle risk factors and relevance to Bangladesh. Diagnosis was solely based on self reporting, details such as symptoms, signs, or lab tests were not collected. The purpose of this study was not to diagnose patients or identify disease based on symptoms.

Providers were further grouped as MBSS (MBBS generalists and MBBS specialist) the expectation was that MBBS doctors all possessed valid medical licenses and practiced allopathic medicine exclusively. Other Qualified Allopathic Practitioners (Other QAP) included nurses, community health workers (government and nongovernment), medical assistants or sub assistant community medical officers. These providers, while not licensed physicians, did possess formal training and qualifications from recognized institutions. Informal Allopathic Practitioners (IAP) (village doctors and medicine shopkeepers) were those practicing allopathic medicine but were not qualified to diagnose or treat patients. Non Allopathic Practitioners (NAP) (Kabiraj/spiritual healers and homeopaths) were grouped as all those not practicing allopathic medicine (formally or informally). The IAP category was provided based on definitions from previous research [21,22,29-32]. Further description of provider plurality in Bangladesh public and private health sector can be found elsewhere [11,12,33].

Descriptive statistics, univariate and multivariate regression analyses were preformed on the study population. The study analyzed disease diagnoses, not the individuals with the disease diagnoses. Odds ratios compared the two largest (proportionally) groupings of providers making chronic disease diagnoses (MBBS doctors and IAPs). Weights were adjusted for cluster effects (w_i _* ∑w_i_/∑w_i_^2^), ie same respondent reporting more than one chronic condition.[34] The age sex distribution of the risk factor study population was adjusted (re-weighted) to be similar to the relative age-sex distribution of the total surveillance populations. All reported statistics were weighted appropriately. SAS (Version 8) statistical software was used to perform the analysis.

## Results

Of the 32,665 survey respondents, 8,591 self reported chronic diseases, 2,907 were urban and 44.9% male while 5,233 were rural and 42.9% male. The mean age was 43.9 years (SD 12.1) (urban) and 51.5 years (SD 14.3) (rural), mean education was 6.9 years (SD 5.1) (urban) and 3.6 (SD 3.9) (rural). 26.2% of urban respondents had no education versus 43.8% rural. 15% of urban respondents had more than secondary education (>10 years) versus only 1.8% of rural. In both urban and rural settings frequency of reporting chronic conditions increased as poverty quintiles increased from most poor to least poor (Table 1).

**Table 1:** Sociodemographic Variables

Categorical Variable (%)*†	Urban n	>Rural n
Total Population	2,907 (36.5%)	5,233 (63.5%)
**Gender**		
Male	1,305 (44.9%)	2,243 (42.9%)
**Education**		
No Education	762 (26.2%)	2,292 (43.8%)
Primary (1-5 years)	481 (16.6%)	1,423 (27.2%)
Secondary (6-10 years)	933 (32.1%)	1,269 (24.3%)
Higher Secondary Education (11-12 years)	294 (10.1%)	153 (2.9%)
Higher Education (>12 years)	437 (15.0%)	96 (1.8%)
**Poverty**‡		
Most poor	265 (9.1%)	568 (11.0%)
More poor	323 (11.1%)	768 (14.8%)
Middle	468 (16.1%)	980 (18.9%)
Less poor	691 (23.8%)	1,200 (23.1%)
Least poor	1,160 (39.9%)	1,669 (32.2%)
**Continuous Variable M (SD)**		
Age	43.9 (12.1)	51.5 (14.3)
Education	6.9 (5.1)	3.6 (3.9)

M- Mean SD-Standard Deviation

* P value < 0.05 considered statistically significant, all values were statistically significant

†Denominator value is respondent not disease

‡ Poverty quintiles identify the proportion of those self reporting a chronic disease by the which poverty quintile they belonged to. Poverty quintiles are representative of the overall population of study respondents (32,665)

Hypertension was the most prevalent self reported chronic disease diagnosis, (21.8% urban versus 16.1% rural). Prevalence of diagnoses differed significantly between urban and rural areas except for stroke, oral cancer and lung cancer. Urban patients with a chronic disease diagnosis were more likely to report additional morbidity. Patients most likely to report additional morbidity in urban areas had dyslipidemia (88.8%), oral cancer (100%) or stroke (85.2%). Patients most likely to report additional morbidity in rural areas had dyslipidemia (78.1%), heart attack (75.5%) or stroke (65.9%) (Table 2).

**Table 2:** Prevalence† of disease diagnoses and comorbidity

	Chronic Condition			Co-morbidity prevalence among patients with specific chronic disease diagnoses		
Prevalence	Urban	Rural	Urban v. Rural	Urban	Rural	Urban v. Rural
Variable n (%)*						
Chronic Disease	2,907 (32.0%)	5,233 (21.8%)	< 0.0001	1,097 (37.7%)	1,047 (20.0%)	< 0.0001
Hypertension	1,463 (16.1%)	2,975 (12.4%)	< 0.0001	767 (52.5%)	848 (28.5%)	< 0.0001
Diabetes	720 (7.9%)	770 (3.2%)	< 0.0001	512 (71.1%)	351 (45.6%)	< 0.0001
Abnormal blood lipids	451 (5.0%)	33 (0.1%)	< 0.0001	400 (88.8%)	26 (78.1%)	0.0664
Overweight	650 (7.2%)	51 (0.2%)	< 0.0001	466 (71.7%)	34 (67.4%)	0.5108
Chronic bronchitis	136 (1.5%)	142 (0.6%)	< 0.0001	60 (44.2%)	36 (25.1%)	0.0008
Heart attack	115 (1.3%)	75 (0.3%)	< 0.0001	85 (75.1%)	57 (75.5%)	0.8211
Coronary heart disease	549 (6.0%)	1,127 (4.7%)	< 0.0001	268 (48.9%)	440 (39.0%)	0.0001
Stroke	167 (1.8%)	444 (1.9%)	0.9479	142 (85.2%)	292 (65.9%)	< 0.0001
Asthma	453 (5.0%)	845 (3.5%)	< 0.0001	193 (42.7%)	210 (24.8%)	< 0.0001
Oral cancer	5 (0.1%)	15 (0.1%)	0.7031	5 (100%)	5 (33.7%)	0.0138
Lung cancer	4 (0.0%)	11 (0.1%)	0.8581	2 (43.1%)	2 (17.4%)	0.3109

MBBS- Medical Bachelors Bachelor of Surgery; Other QAP- Other Qualified Allopathic Provider; IAP Informal Allopathic Provider; NAP- Non-Allopathic Provider

* P value < 0.05 considered statistically significant, all values were statistically significant

†Prevalence denominator was based on respondent

‡ Provider diagnosis proportion denominator was self reported disease

MBBS doctors were most frequently reported as the providers making the most recent diagnosis for every disease, and were reported more frequently in urban than rural Bangladesh for every disease except coronary heart disease and lung cancer. IAP were the second most commonly identified as providing the most recent diagnosis in every category except for urban asthma diagnoses (18% NAP). NAP and Other QAP contributed a nominal proportion of all other diagnoses (Table 3).

**Table 3:** Proportion of Chronic Disease by Diagnosing Providers

	Proportion of Diagnoses‡							
	MBBS		Other QAP		IAP		NAP	
	urban	rural	urban	rural	urban	rural	Urban	rural
Variable (%)*								
Chronic Disease	3990 (88.0%)	3605 (59.7%)	117(2.6%)	158 (2.6%)	357 (7.9%)	2107 (34.9%)	71 (1.6%)	165 (2.7%)
Hypertension	1300 (89.0%)	1407 (47.4%)	29 (2.0%)	94 (3.2%)	124 (8.5%)	1,443 (48.6%)	8 (0.6%)	26 (0.9%)
Diabetes	698 (97.4%)	670 (87.0%)	7 (0.9%)	16 (2.1%	11 (1.5%)	64 (8.3%)	2 (0.2%)	20 (2.5%)
Abnormal blood lipids	446 (99.0%)	28 (86.4%)	1 (0.2%)	0 (0.0%)	2 (0.6%)	4 (13.6%)	0 (0.0%)	0 (0.0%)
Overweight	626 (96.4%)	45 (88.9%)	9 (1.3%)	1 (1.5%)	9 (1.5%)	4 (.1%)	5 (0.9%)	1 (1.5%)
Chronic bronchitis	108 (79.2%)	89 (63.1%)	3 (2.3%)	2 (1.7%)	20 (14.4%)	37 (26.4%)	6 (4.2%)	13 (8.9%)
Heart attack	111 (97.8%)	66 (88.9%)	0 (0.0%)	0 (0.0%)	2 (2.2%)	8 (11.1%)	0 (0.0%)	0 (0.0%)
Coronary Heart disease	376 (68.5%)	879 (78.0%)	17 (3.2%)	32 (2.8%)	143 (26.1%)	168 (14.9%)	12 (2.2%)	48 (4.3%)
Stroke	160 (95.9%)	319 (71.8%)	3 (1.6%)	5 (1.1%)	0 (0.0%)	102 (23.0%)	4 (2.5%)	18 (4.2%)
Asthma	319 (70.9%)	402 (47.6%)	5 (1.2%)	19 (2.3%)	42 (9.4%)	372 (44.1%)	84 (18.6%)	50 (6.0%)
Oral cancer	4 (83.1%)	9 (64.4%)	0 (0.0%)	0 (0.0%)	1 (16.9%)	5 (35.6%)	0 (0.0%)	0 (0.0%)
Lung cancer	2 (62.2%)	10 (86.3%)	0 (0.0%)	1 (6.9%)	1 (36.8%)	1 (6.8%)	0 (0.0%)	0 (0.0%)

MBBS- Medical Bachelors Bachelor of Surgery; Other QAP- Other Qualified Allopathic Provider; IAP Informal Allopathic Provider; NAP- Non-Allopathic Provider

* P value < 0.05 considered statistically significant, all values were statistically significant

†Prevalence denominator was based on respondent

‡ Provider diagnosis proportion denominator was self reported disease

MBBS doctors were the most frequently (IAP second most) identified as being the healthcare provider to make the most recent diagnosis by all sociodemographic groups except *most poor*. A higher proportion of women (39.1%) than men (29.2%) relied on IAP in rural areas. IAP in rural areas provided more diagnoses (56.6%t) to *most poor *respondents compared MBBS doctors (36.4%). Other QAP and NAP comprised a comparatively nominal contribution to diagnoses in all groups. The proportion of diagnoses made by MBBS doctors increased with increasing education and decreasing poverty in both urban and rural environments (Table 4).

**Table 4:** Proportion‡ of most recent diagnostic provider across sociodemographic groups

	MBBS		IAP		NAP		Other QAP	
	urban	rural	urban	rural	urban	rural	urban	rural
Variables (%)*†								
**Total**	3990 (88.0%)	3605 (59.7%)	357 (7.9%)	2107 (34.9%)	117(2.6%)	158 (2.6%)	71 (1.6%)	165 (2.7%)
**Gender**								
Male	1777 (88.0%)	1691 (65.7%)	155 (7.7%)	753 (29.2%)	64 (3.2%)	81 (3.1%)	23 (1.1%)	50 (1.9%)
Female	2214 (88.0%)	1914 (55.3%)	201 (8.0%)	1354 (39.1%)	53 (2.0%)	77 (2.2%)	48 (1.9%)	115 (3.3%)
**Age**								
25 - 40	1360 (83.1%)	775 (56.5%)	189 (11.6%)	481 (35.1%)	53 (3.4%)	45 (3.3%)	34 (2.1%)	71 (5.2%)
40 - 50	1188 (89.9%)	791 (58.3%)	82 (6.2%)	493 (36.4%)	31 (2.4%)	43 (3.2%)	21 (1.6%)	29 (2.1%)
50 - 60	903 (91.0%)	916 (63.2%)	62 (6.3%)	468 (32.6%)	18 (1.7%)	35 (2.4%)	9 (0.9%)	31 (2.1%)
60+	540 (92.6%)	1123 (60.5%)	22 (3.8%)	664 (35.8%)	14 (2.4%)	36 (1.9%)	7 (1.2%)	34 (1.8%)
**Education**								
No education	839 (79.7%)	1236 (48.9%)	152 (14.4%)	1153 (45.6%)	38 (3.5%)	53 (2.1%)	24 (2.3%)	84 (3.3%)
Primary	614 (83.9%)	1063 (66.6%)	74 (10.1%)	519 (31.0%)	28 (3.8%)	49 (2.9%)	16 (2.2%)	41 (2.5%)
Secondary	1410 (91.5%)	1052 (69.3%)	82 (5.3%)	389 (25.6%)	26 (1.8%)	43 (2.8%)	23 (1.5%)	34 (2.2%)
Higher secondary	426 (91.6%)	159 (81.1%)	25 (5.4%)	27 (13.8%)	9 (2.1%)	8 (4.1%)	5 (1.1%)	2 (1.0%)
Higher	700 (94.3%)	95 (77.2%)	24 (3.2%)	19 (15.4%)	15 (2.0%)	5 (4.1%)	3 (0.4%)	4 (3.3%)
**Poverty status**								
Most poor	186 (60.2%)	215 (36.4%)	97 (31.4%)	334 (56.6%)	17 (5.6%)	16 (2.7%)	9 (2.9%)	25 (4.2%)
More poor	392 (73.6%)	385 (46.7%)	65 (16.4%)	380 (46.1%)	29 (7.2%)	28 (3.4%)	11 (2.8%)	32 (3.9%)
Middle	611 (87.9%)	616 (55.2%)	56 (8.1%)	438 (39.2%)	15 (2.2%)	34 (3.0%)	13 (1.9%)	28 (2.5%)
Less poor	957 (90.1%)	817 (60.3%)	62 (5.8%)	464 (34.2%)	24 (2.3%)	35 (2.6%)	19 (1.8%)	40 (2.9%)
Least poor	1950 (93.9%)	1538 (76.6%)	76 (3.7%)	467 (22.4%)	31 (1.5%)	45 (2.2%)	19 (0.9%)	39 (1.9%)

MBBS- Medical Bachelors Bachelor of Surgery; Other QAP- Other Qualified Allopathic Provider; IAP Informal Allopathic Provider; NAP- Non-Allopathic Provider

* P value < 0.05 considered statistically significant, all provider group values were statistically significant compared to each other.

†All figures are based on disease diagnoses not respondents with disease

After adjusting for covariates, men in rural areas were shown to have higher odds (1.35) than women in rural areas of seeking care from an MBBS doctor versus an IAP. As poverty decreased, odds of an MBBS doctor making the most recent diagnosis increased. Odds of receiving the latest diagnosis from an MBBS doctor increased with increasing age in urban areas. This effect was not seen in rural areas. Odds of reporting a diagnosis made by an MBBS doctor shared a nonlinear relationship with increasing education (Table 5).

**Table 5:** Odds ratios of Care Seeking MBBS versus IAP care*

	Urban Multivariate (n = 4347)			Rural Multivariate (n = 5712)		
	OR	CI	p-value†	OR	CI	p-value†
**Sex **(male = 1)	0.83	(0.64-1.06)	0.13	1.35	(1.18-1.55)	<0.001
**Comorbidity**	2.12	(1.67-2.70)	<0.001	2.99	(2.62-3.40)	<0.001
**Age **(in years)						
25 - 39 (reference)						
40-49	1.86	(1.40-2.47)	0.77	0.88	(0.74-1.05)	0.35
50-59	1.88	(1.35-2.60)	0.74	0.99	(0.83-1.18)	0.17
60+	3.03	(1.90-4.82)	0.002	0.84	(0.71-0.99)	0.05
**Education**						
No education (reference)						
Primary (1-5 years)	1.25	(0.91-1.71)	0.03	1.49	(1.30-1.72)	0.15
Secondary (6-10 years)	1.99	(1.42-2.79)	0.12	1.57	(1.33-1.85)	0.37
Higher Secondary (11-12 years)	1.81	(1.07-3.05)	0.65	2.78	(1.75-4.42)	0.01
Higher Education	2.81	(1.65-4.80)	0.006	2.15	(1.21-3.82)	0.30
**Poverty status**						
Most poor (reference)						
More poor	2.10	(1.46-3.02)	0.005	1.35	(1.08-1.70)	<0.001
Middle	4.17	(2.86-6.09)	0.01	1.86	(1.50-2.30)	0.73
Less poor	4.53	(3.07-6.67)	0.006	2.22	(1.80-2.75)	0.003
Least poor	6.10	(4.11-9.06)	<0.001	3.57	(2.89-4.42)	<0.001

MBBS- Medical Bachelor Bachelor of Surgery, IAP- Informal Allopathic Practitioner, OR-Odds Ratio,

CI-Confidence Interval

*Odds ratios are based on disease diagnoses, not respondents with disease

†p-value represents the significance of the difference from the aggregation of the remaining variable

## Discussion

### Main Conclusion

IAPs are a major provider of most *recent *chronic disease diagnoses in urban and rural areas. Although IAPs provide as much as 2/3 of first line care in Bangladesh [15], MBBS doctors comprise the largest proportion of most recent chronic disease diagnoses. Previous acute and primary care research that focuses on providers who make the *initial *diagnoses indicate that IAPs provide a larger portion of those *initial *diagnoses [11-14,29,30,35,36]. Chronic diseases require long term management for symptoms that do not resolve easily. In seeking care for a protracted illness, chronic disease patients are likely to receive multiple diagnoses and consultations from the plurality of providers in Bangladesh. IAPs are usually the point of first contact in rural areas, and make diagnoses primarily based on patient symptoms [12,17,35,37]. When symptomatic, chronic diseases are typically advanced and complex; IAPs are known by Bangladeshis as quick accessible resources for simple acute disease [18]. Once the disease is deemed too complex for the IAP's skills, patients often seek or are referred to MBBS doctors for more specialized care [11,18]; IAP may make a higher number of initial chronic disease diagnoses [20]. Alternatively, care seekers may perceive chronic disease symptoms as severe enough to initially seek care from an MBBS doctor, despite the cost and inconvenience [18]. However, such behavior is not seen in the care seeking patterns around other common diseases and is a less likely explanation of our results [18,35,38]. As first line care providers, IAP encounter chronic diseases before MBBS doctors, what is unknown is if they correctly diagnose these diseases, how they diagnose them and what steps they take afterwards.

### Subconclusion: Healthcare provider differences in urban and rural Bangladesh

There were very clear differences between urban and rural chronic disease diagnoses throughout the study. Inequalities between urban and rural access to MBBS doctors leave an estimated 75% of Bangladesh's population with substandard healthcare options [39]. There is a lack of doctors working in rural areas with at least 26% of rural posts unfilled and absenteeism as high as 74% [11,40]. There are an estimated 1.1 physicians per 10,000 population in rural Bangladesh, 18.2 physicians in urban areas.[11] A number of disincentives and lack of incentives makes filling these posts extremely difficult [40]. The resulting lack of access to rural MBBS doctors was strongly reflected throughout our study results. Transportation expenses, logistics and hidden service costs further compound the difficulties of accessing healthcare from an MBBS doctor [12,18]. The more developed urban infrastructure makes transportation less of an obstacle to seeking high quality care. The issues surrounding accessibility are particularly difficult in chronic diseases where proper disease management require routine visits. Bangladesh cannot manage the chronic disease epidemic through MBBS doctors alone. Given the urban and rural inequalities in accessing MBBS doctors, the support of NGO's, community health workers and those making the disease diagnoses, like IAP, will be essential to controlling the chronic disease epidemic. However, IAP may not be fully prepared to manage these diseases in a manner that prevents their disabling chronic disease sequela. A better understanding of current IAP knowledge and practice regarding chronic disease will be essential to shaping their potential role as formal sector collaborators.

### Vulnerable groups seeking chronic disease care

Rural IAPs serve vulnerable hard to service groups, making them incredibly important to providing health coverage. Most Bangladeshis in the "most poor" quintile reported IAPs as the healthcare provider who diagnosed their disease most recently, not MBBS doctors. Poverty places these patients at increased risk of chronic disease morbidity, that burden is compounded by the unknown and likely questionable practices of Bangladeshi IAPs. Most poor also had the lowest rate of diagnoses; this could be explained by poverty and reluctance to seek diagnoses requiring unaffordable treatments. In rural areas, being a woman significantly increased one's odds of having a diagnosis made by an IAP versus an MBBS doctor; this effect was not seen in urban settings. Gender inequality is a major problem in disease care seeking; women report more disease, but seek care from MBBS less frequently than men [18]. Gender inequality in care seeking is often attributed to a lack of autonomy; usually a male household head makes care seeking decisions for the woman [18]. Cultural pressure to maintain purdah, compounded by a lack of female providers further inhibits women with chronic disease from seeking care from MBBS doctors. Purdah is the cultural practice of maintaining modesty (i.e. clothing women and secluding them from males who are not blood relatives) [19,38,41]. Purdah can make a few kilometres to the doctor's office an impossible distance for an unescorted woman. Some studies have suggested that urbanization may decrease healthcare gender inequalities [42]. Accessibility of the physician in urban areas is made possible by several factors: 1) higher urban physician (especially female physician) population density ratios 2) reduced travel time to healthcare facilities 3) superior transportation infrastructure 4) denser populations with stronger social support networks that maintain purdah without restricting mobility [11,12,43,44]. Developing effective health policy to manage the chronic disease epidemic must take precautions to protect vulnerable groups and address the challenges they face accessing care.

There were several strengths in this study. The size and geographic reach of this study provide some initial steps in providing more nationally representative data. The size of our study also helps to support the accuracy of our findings in these areas. By describing the chronic disease epidemic within the context of Bangladesh's pluralistic healthcare system we are able to provide a more accurate picture of care seeking than studies that simply focus on care provided by the formal sector. Examining which providers most recently diagnosed a patient's illness gives more indication as to where care seekers go for conditions requiring longer term management [20]. This is especially important when looking at chronic diseases, which are far more likely to involve multiple providers along the course of the disease.

There are some important limitations in this study. Validity of diagnosis is an obvious concern; however the focus of this paper was prevalence and source of diagnoses, not disease prevalence. Research aimed at establishing diagnostic validity and more accurate disease prevalence will require a research team of trained professionals examining only a few measurable diseases (ie hypertension). Although self reported disease status has long been used as an epidemiological tool, there is an inherent risk of bias by omission (intentional or accidental) of actual care seeking behaviors. Though reporting bias is a concern, HDSS routine surveillance increases the populations' familiarity with questionnaires and interviewers, decreasing the likelihood of reporting bias. Furthermore, there may be a great deal of undiagnosed chronic disease in this population for which people are seeking symptomatic treatment. While the study is a large scale study, we cannot yet assert that it is fully representative of the national scenario in Bangladesh. The HDSS was originally designed to collect data on child and maternal health. Men typically work during the day when HDSS teams visit the households, excluding some men from the sampling. This skewed the population towards women. It is unknown if the care seeking of those at home during the time of interview differs significantly from those not at home. While this bias was adjusted for, alternative data collection strategies need to be deployed to gather surveillance data on health issues affecting working adults, particularly men.

Both MBBS doctors and IAPs interact with the chronically ill but not necessarily at the same point in the care seeking process. IAPs are more likely to encounter the chronically ill early in the disease process when symptoms may be too subtle for them to accurately diagnose or even detect. The IAPs have a crucial opportunity to catch chronic diseases early but will likely need training and support to screen for such diseases. Currently, large numbers of chronically ill Bangladeshis may receive inadequate care for their diseases due to lack of MBBS doctor accessibility and gaps in IAPs' knowledge and practice [20,45,46]. Rural areas, women and the most poor are at particular risk of these complications. Improving chronic disease management will require a more thorough understanding of how Bangladeshis with chronic diseases seek diagnoses. Establishing linkages between MBBS doctors and IAPs creates opportunities for better quality chronic disease prevention, screening, and management, among a larger proportion of the population. Broader coverage of better quality will reduce potential economic and social impacts of a chronic disease epidemic.

Our study confirms that IAPs play a significant role diagnosing chronic disease. The quality of these diagnoses, subsequent treatments and the extent to which IAPs provide treatment remains unknown. Previous research indicates IAP are likely to have substandard knowledge and practice regarding chronic diseases [12,32,47-50]. While MBBS doctors are the considered to be the highest level of care available, literature on their knowledge and practice regarding chronic diseases is lacking in Bangladesh. Previous studies in Pakistan indicate that Bangladeshi MBBS doctors may not be managing chronic diseases in accordance with international guidelines [51]. Further research of MBBS doctors' chronic disease knowledge and practice is needed to assess the formal sector's preparedness for this epidemic. IAPs are already entrenched in the communities they serve, where they are relied on for accessible healthcare coverage. Chronic disease knowledge and practice studies are needed to further evaluate the potential role IAP can play in the formal sector's efforts to control the chronic disease epidemic.

## Conclusion

IAP are crucial to properly managing the chronic disease epidemic in Bangladesh and will continue to be so. As the healthcare system catches up to the countries disease epidemiology, it will be important to enact policies to ensure that future generations of IAP are prepared for this shift. Based on the findings in this study, research should be undertaken to better evaluate the knowledge and practice of rural IAP regarding common chronic diseases. Given the lack of MBBS doctors in rural areas, efforts should focus on developing linkages between IAP and MBBS doctors to improve the quality of care accessible to Bangladesh's mostly rural population. Health issues most pressing to vulnerable groups like the rural poor and rural women should be further researched and subsequent findings used to improve the specific care needs among this population.

## Competing interests

The authors declare that they have no competing interests.

## Authors' contributions

JP was involved in this study's conception and design, analysis and interpretation of data, and drafting the manuscript. WL was involved in this study's conception and design, acquisition of data, analysis and interpretation of data, and revising the manuscript. MK was involved in this study's conception and design, acquisition of data, analysis and interpretation of data, and revising the manuscript. TK was involved in this study's conception and design, analysis and interpretation of data, supervising the research team and revising the manuscript. All authors read and approved the final manuscript.

## Pre-publication history

The pre-publication history for this paper can be accessed here:

http://www.biomedcentral.com/1472-6963/11/309/prepub

## Supplementary Material

Additional file 1 Additional file 1** contains the cross sectional survey used to obtain study data**. Click here for file
