# Supplementary material for: Diagnosis of chronic conditions with modifiable lifestyle risk factors in selected urban and rural areas of Bangladesh and sociodemographic variability therein
Source: BMC Health Serv Res. 2011 Nov 11;11:309. doi: 10.1186/1472-6963-11-309 (PMC3239323; doi:10.1186/1472-6963-11-309)
Supplement: Additional file 1 — contains the cross sectional survey used to obtain study data. [file 1472-6963-11-309-S1.DOC]

Questions on risk factors for Chronic Diseases:

CID: _ _ _ _ _ _ _ _ _ . HH: _ _ _ _ _ _ _, ID: _ _.

RID: _ _ _ _ _ _ _ _ _ _. PID: _ _ _ _ _ _ _ _ _.

1. Physical activities:

**a. How many times a week you usually do for 20 minutes or more vigorous-intensity physical activity (eg heavy lifting, digging, boat driving or rickshaw pulling) that makes you sweat or puff and pant?**

a. >3 times/ week

b. 1-2 times/ week

c. none

**b. How many times a week do you usually do for 30 minutes or more moderate-intensity physical activity or walking (eg carrying light loads, bicycling at a regular pace, cleaning the house or washing cloths) that increases your breathe faster than normal?**

a. >5 times/ week

b. 3-4 times/ week

c. 1-2 times/ week

d. none

**3.** **How many cups of fresh or cooked vegetables did you have yesterday?___**

**4.** **How many cups of fruit did you have yesterday?_____**

**5.** **Do you smoke daily (Filter tipped cigarette/ Biri/ Hukka)?**

1. Yes 2. No (If no go to Q. no 6)

**5.1** **For how long have you been smoking?**  ____ months/ years

**5.2 How many sticks daily?** ____ (Go to Q. no. 7)

**6.** **Have you smoked before?** 1. Yes 2. No (If no go to Q. no 7)

**6.1** **For how long?**  __ months/ years

**7.** **Do you take betel or tobacco leaf** **daily?**  1. Yes 2. No (If no go to Q. no 8)

**7.1** **For how long have you been taking betel or tobacco leaf?** ___months/years

**7.2 How many times daily?** ____ (Go to Q. no. 9)

**8.** **Have you taken betel or tobacco leaf before?** 1. Yes 2. No (If no go to Q. no 9)

**8.1** **For how long?**  __ months/ years

| **9. Have you ever been told by any of the following personnel**  1. MBBS doctor  2. Specialized doctor  3. Nurse  4. Health worker  5. Paramedic (MA/SACMO)  6. Village doctor/Quack  7. Homeopath  8. Kabiraj  9. Pharmacy man  **that you have any of the following medical conditions?** | | **Diagnose**  Yes= 1  No= 2 | Last Diagnosed by  (*write the code*)  Not applicable=88 | Any treatment received?  Yes= 1  No= 2  Not applicable=88 | **Mode of treatment:**  1. Medicine  2. Hospital admission  3. Surgery  4. Dietary advice  5. Health education  6. Exercise  7. Other___  *(Multiple response possible)* | Type of provider:  01. MBBS doctor  02. Specialized doctor  03. nurse  04. Health worker  05. Paramedic (MA/SACMO)  06. Village doctor/Quack  07. Homeopath  08. Kabiraj  09. Pharmacy man  10. Self  11. Other__  88. Not applicable  *(Multiple response possible)* | Are you currently under treatment?   1. No 2. Medicine 3. Diet 4. Exercise 5. Others____   88. Not applicable |
| --- | --- | --- | --- | --- | --- | --- | --- |
| **9a** | **Hypertension** |  |  |  |  |  |  |
| **9b** | **Diabetes** |  |  |  |  |  |  |
| **9c.** | **Abnormal blood lipids** |  |  |  |  |  |  |
| **9d** | **Overweight** |  |  |  |  |  |  |
| **9e** | **Chronic bronchitis** |  |  |  |  |  |  |
| **9f** | **Heart attack** |  |  |  |  |  |  |
| **9g** | **Angina or coronary heart disease** |  |  |  |  |  |  |
| **9h** | **Stroke** |  |  |  |  |  |  |
| **9i** | **Asthma** |  |  |  |  |  |  |
| **9j** | **Oral Cancer** |  |  |  |  |  |  |
| **9k** | **Lung cancer** |  |  |  |  |  |  |
| **9l** | **Other 1** |  |  |  |  |  |  |
| **9m** | **Other 2** |  |  |  |  |  |  |
